# Supplementary figures and images for: Interpopulation morphological differences and sexual dimorphism of Dekay’s brownsnake (Storeria dekayi) along a rural–urban gradient
Source: PeerJ. 2025 Jun 11;13:e19439. doi: 10.7717/peerj.19439 (PMC12166847; doi:10.7717/peerj.19439)

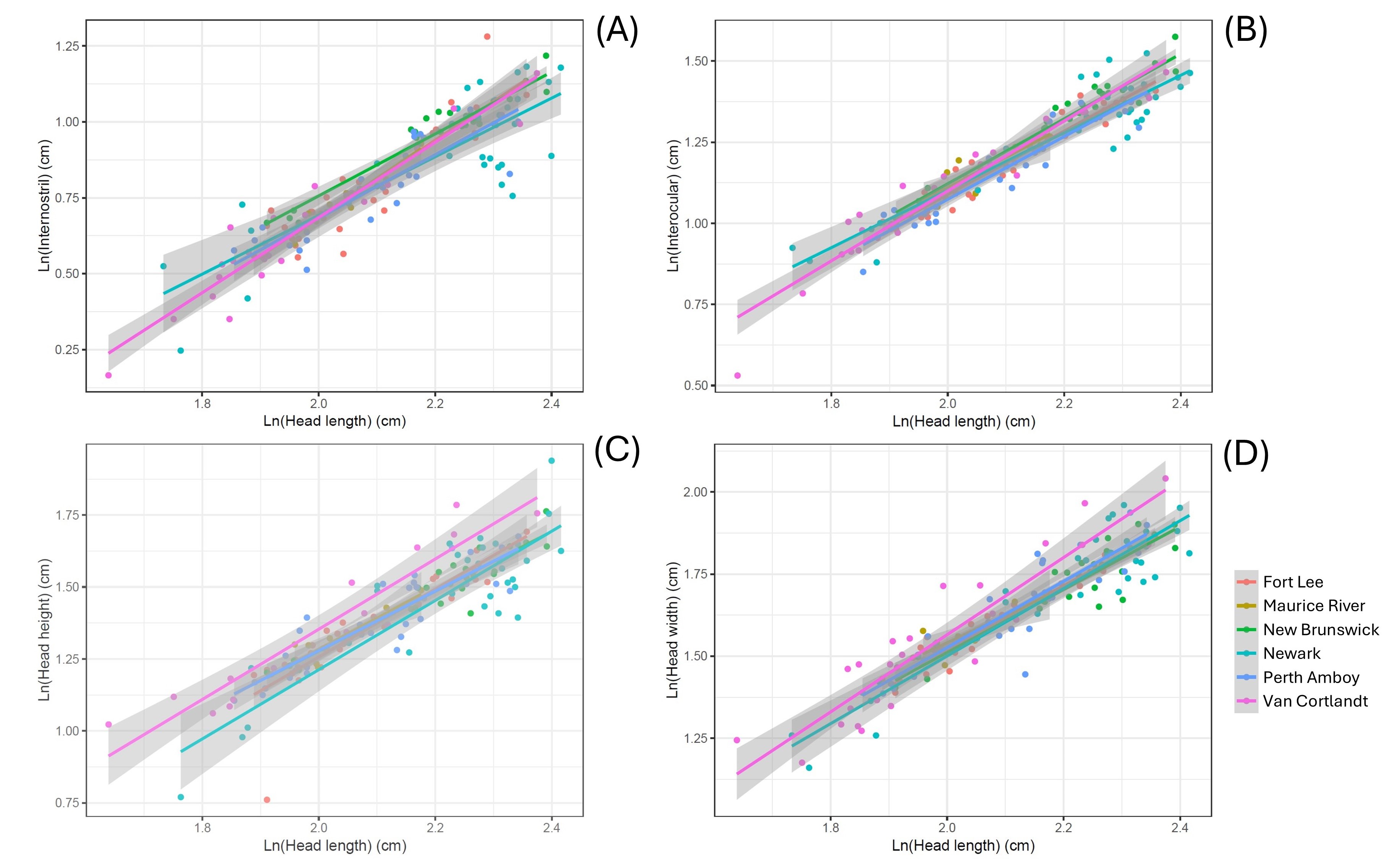

Supplement: Supplemental Information 1 — (A) Internostril (F = 2.680, P = 0.024), (B) interocular (F = 3.530, P = 0.005), (C) head height (F = 4.092, P = 0.002), and (D) head width (F = 2.833, P = 0.018). [file peerj-13-19439-s001.jpg]

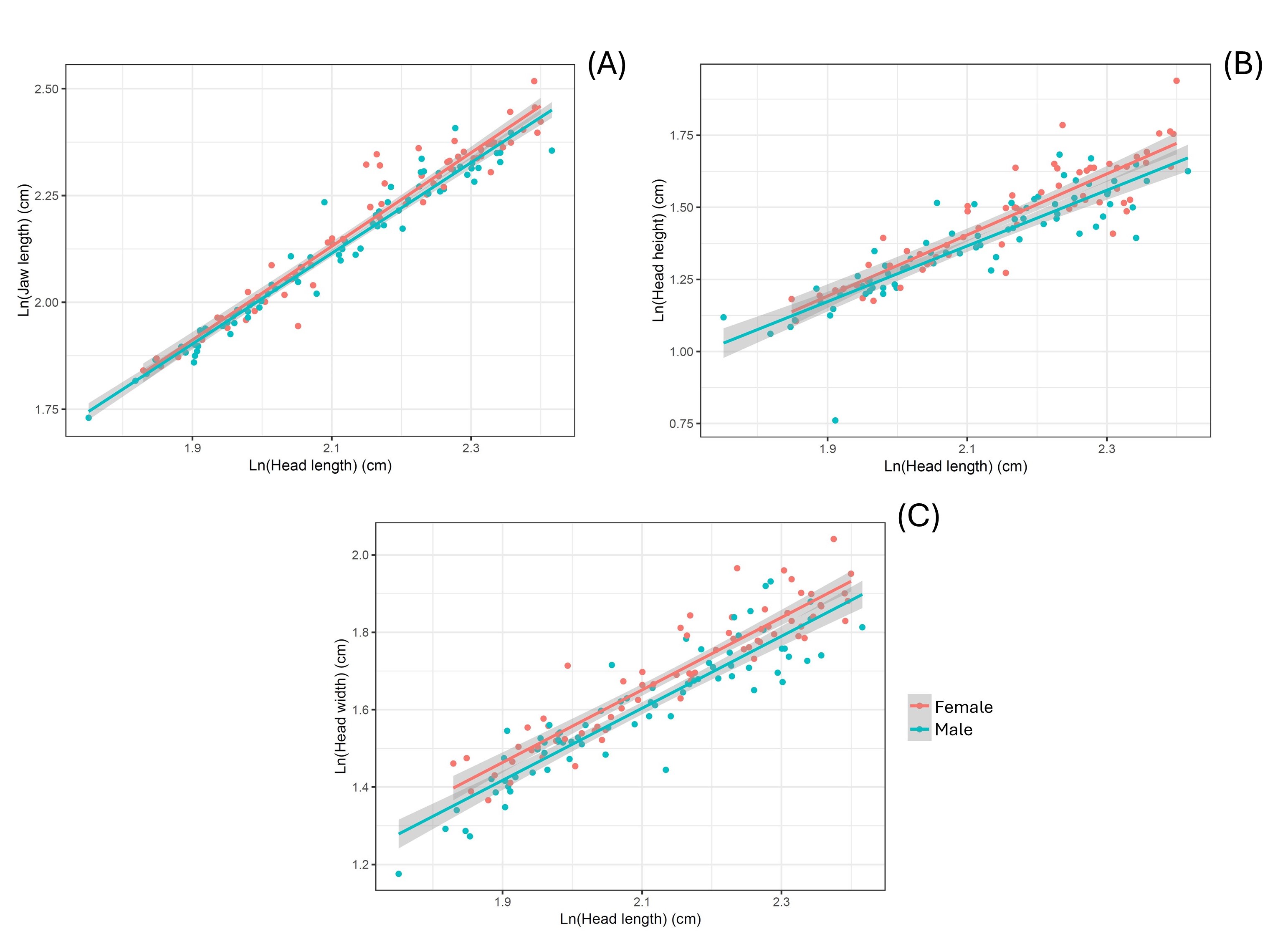

Supplement: Supplemental Information 2 — Females have (A) a longer jaw (F = 6.482, P = 0.012), (B) a larger head height (F = 7.797, P = 0.006), and (C) a wider head (F = 19.415, P < 0.001) than males. [file peerj-13-19439-s002.jpg]
